# Implementing Internet-Based Self-Care Programs in Primary Care: Qualitative Analysis of Determinants of Practice for Patients and Providers

**DOI:** 10.2196/mental.9600

**Published:** 2018-05-18

**Authors:** Eric Hermes, Laura Burrone, Elliottnell Perez, Steve Martino, Michael Rowe

**Affiliations:** ^1^ VA Connecticut Healthcare System Veterans Health Administration West Haven, CT United States; ^2^ Department of Psychiatry Yale University School of Medicine New Haven, CT United States

**Keywords:** cognitive behavioral therapy, internet-based therapy, health information technology, behavioral intervention technology, internet, Veterans

## Abstract

**Background:**

Access to evidence-based interventions for common mental health conditions is limited due to geographic distance, scheduling, stigma, and provider availability. Internet-based self-care programs may mitigate these barriers. However, little is known about internet-based self-care program implementation in US health care systems.

**Objective:**

The objective of this study was to identify determinants of practice for internet-based self-care program use in primary care by eliciting provider and administrator perspectives on internet-based self-care program implementation.

**Methods:**

The objective was explored through qualitative analysis of semistructured interviews with primary care providers and administrators from the Veterans Health Administration. Participants were identified using a reputation-based snowball design. Interviews focused on identifying determinants of practice for the use of internet-based self-care programs at the point of care in Veterans Health Administration primary care. Qualitative analysis of transcripts was performed using thematic coding.

**Results:**

A total of 20 physicians, psychologists, social workers, and nurses participated in interviews. Among this group, internet-based self-care program use was relatively low, but support for the platform was assessed as relatively high. Themes were organized into determinants active at patient and provider levels. Perceived patient-level determinants included literacy, age, internet access, patient expectations, internet-based self-care program fit with patient experiences, interest and motivation, and face-to-face human contact. Perceived provider-level determinants included familiarity with internet-based self-care programs, changes to traditional care delivery, face-to-face human contact, competing demands, and age.

**Conclusions:**

This exploration of perspectives on internet-based self-care program implementation among Veterans Health Administration providers and administrators revealed key determinants of practice, which can be used to develop comprehensive strategies for the implementation of internet-based self-care programs in primary care settings.

## Introduction

### Background

Internet-based self-care programs (ISPs) belong to a category of health information technology, which consist of personalized, self-guided interventions delivered electronically to improve knowledge, awareness, or behavior change for mental or behavioral health conditions [1]. ISPs present interactive therapeutic material in rich formats such as audio, video, and text. Participants use ISPs at a pace and in a setting of their choosing, while being provided varying levels of support [2,3]. Evidence-based ISPs have shown efficacy for the treatment of many common conditions including depression, anxiety, substance use, and insomnia [4,5].

The potential health service benefits of ISPs are pronounced, as they may mitigate chronic and systemic problems associated with access to mental health services [6]. Access to services is a challenge even for integrated health care systems such as Veterans Health Administration (VHA), which are accountable to provide a coordinated range of services to a defined population [7,8]. One solution may lie in the implementation of ISPs, the benefits of which include reduced travel barriers, improved access to evidence-based therapies, and reduced stigma [9]. ISPs may also increase self-care and add to health care system productivity [10,11]. Within the VHA, ISPs may especially benefit patients where travel time and distance to VHA facilities or the lack of trained providers are limiting factors [12].

Despite the advantages of ISPs and evidence of general acceptability, ISP use in US health care systems, including the VHA, is not well documented and presumed low [13,14]. Although ISP use in the United Kingdom, Europe, and Australia is more routine and to some extent institutionalized in policy, there have been notable difficulties with ISP implementation at the point of care with patient and providers in the primary care settings [15-17]. For instance, a recent major pragmatic trial implementing ISPs for depression in primary care sites across the United Kingdom resulted in relatively low use of the programs by patients [18].

The lack of ISP use in US health care settings and the relative difficulty of implementation in other countries suggest the need for comprehensive strategies to address ISP implementation. Such strategies are dependent on the determinants of practice for a given setting—defined as the human and system factors, which determine to what extent and manner interventions are used. Determinants of practice have been referred to elsewhere by alternative terms such as barriers, obstacles, enablers, and facilitators [19]. For instance, in a study implementing evidence-based practices for the treatment of chronic diseases in primary care, the use of checklists by providers and self-management among patients were identified as important determinants of practice [20]. However, unlike other interventions, determinants of practice for ISPs rely on the acceptance and use of both technology and self-care by patients, providers, staff, and administrators [21].

### Objective

The identification of determinants of practice for ISP use will be a key step in the development of effective strategies for ISP implementation [22]. The objective of this study was to identify determinants of practice for ISP implementation perceived by VHA primary care and integrated mental health care providers and administrators. The identification of determinants will aid in the development of comprehensive strategies for ISP implementation.

## Methods

### Study Design

VHA primary care provider and administrator perspectives on ISP implementation were explored through qualitative analysis of semistructured interviews. The institutional review board of the VA Connecticut Healthcare System preapproved this study.

### Sample

To identify determinants encountered in care, participants were primary care providers, primary care mental health providers, clinic/facility administrators, and VHA system administrators who were known to be using or have used ISPs in patient care, interested in using ISP in primary care settings, or otherwise knowledgeable on the subject of ISP use. VHA does not systematically track or record patient or provider use of ISPs, and as yet, there is no validated way to search VHA electronic health records for evidence of use. Therefore, a provider sampling strategy based on in-depth investigator knowledge of the VHA system was used. Investigators identified an initial group of VHA key informants in ISP use. This group was expanded by asking interviewees for referrals to additional key informants. Recruitment was through e-mail contact that included a project description. Physicians, psychologists, nurse practitioners, nurse administrators, and social workers with both clinical and administrative responsibilities were recruited. A total of 20 interviews were conducted; 100% of those approached agreed to participate.

### Data Gathering

The principal investigator (EH) conducted semistructured interviews using a guide that first defined ISPs, giving examples of VHA-developed ISPs and other programs if needed, such as Moving Forward, PTSD Coach, and Sleep Healthy Using the Internet [23-25]. Determinants were then explored using 9 core question areas, with subquestions to explore specific topics that emerged. Question areas were derived from constructs (performance expectancy, effort expectancy, social influence, and facilitating conditions) from the Unified Theory of Acceptance and Use of Technology, a theory that describes factors associated with technology use [21]. Interviews were audio recorded and conducted over the phone, lasting between 45 and 90 min.

### Data Analysis

Interviews were transcribed verbatim. Thematic analysis, a foundational approach to the analysis of in-depth interviews, was used to analyze interview content through a 5-step group process: (1) familiarization with transcribed data; (2) generation of initial codes; (3) collating codes into themes; (4) reviewing, discussing, and modifying themes in relation to interview extracts; and (5) defining and developing a typology of themes [26]. Codes were first generated by individual investigators, and then discussed as a group, referencing the interview content to develop a final set of codes. Discrepancies in coding were addressed through group discussions led by the principal investigator (EH). Similarly, themes were then developed from codes using a group discussion process. Codes within themes were reviewed using the entire dataset to identify examples and counterfactuals. Authors EH, LB, and EP participated in steps 1 through 3, whereas all authors participated in steps 4 and 5. Themes were organized according to socio-ecological levels within the integrated health care system: patients, providers, and the organization [27]. This manuscript describes patient- and provider-level themes associated with determinants of practice for ISP use at the point of care. Determinants active at the VHA organizational level will be described in future work. Data were managed and analyzed using Atlas/ti. version 4.2 software (ATLAS.ti Scientific Software Development GmbH).

## Results

### Interviewee Characteristics

Of the 20 individuals participating, more males than females were interviewed, while the training, background, and primary work locations of interviewees were relatively balanced (Table 1). Interviews began with questions concerning interviewees’ and their colleagues’ experience with using ISPs in clinical practice. ISP use among interviewees was relatively low. Although 16 (80%, 16/20) interviewees had used an ISP or discussed ISPs with patients at some point in practice, only 6 (30%, 6/20) were currently using them. One primary care provider stated:

I have [used ISPs], but not typically, this isn’t something that’s kind of a normal or typical thing for me to do.

Despite this level of use, all 20 interviewees (100%, 20/20) made statements in support of ISP use in primary care. One primary care provider stated:

I’m all for it. I think that it’s a great modality to utilize especially as we are getting…a lot more of the younger guys…who are much more computer tech savvy.

### Perceived Determinants Associated With Patients

#### Attitude Toward Treatment

A total of 10 interviewees (50%, 10/20) identified patient interest in, motivation for, or willingness to engage in ISPs or mental health treatment in general as a determinant of ISP use. Interest in ISPs was generally described as high, but variable. One primary care provider with administrative responsibilities said most patients are using the internet for health information already:

A lot of them…do a lot of exploration of their problems and try things out.

Interviewees also discussed how patients must adjust to the idea of self-care and are variably motivated to engage in self-care. Consequently, providers assess patient interest and motivation before recommending ISP use. One provider in primary care mental health said:

I meet with the patient and I pull up the course and we go through some of it and I get a sense of whether or not they have the interest in continuing to do it on their own.

Another said:

Motivation is a big one for everything. The big barrier is if they’re not really motivated to do whatever, then they’re likely not going to do it.

**Table 1:** Characteristics of interviewees.

Characteristic		Interviewees (N=20), n (%)
**Gender**		
	Male	13 (65)
	Female	7 (35)
**Training**		
	Doctor of Philosophy	9 (45)
	Medical Doctor	6 (30)
	Registered Nurse	3 (15)
	Social work	2 (10)
**Primary work location**		
	Primary care	8 (40)
	Primary care mental health	8 (40)
	ISP implementation research^a^	4 (20)
**Primary duty^b^**		
	Patient care	12 (60)
	Administration	8 (40)

^a^Interviewees with direct experience with VA patients and providers in VA primary care and primary care mental health settings as part of ISP research.

^b^All interviewees had both clinical and administrative duties. This category identifies their primary duty activity.

#### Literacy

Low technology and reading literacy were discussed in 8 interviews (40%, 8/20), especially in older Veterans. As a clinic administrator stated:

I think that [the use of] technology-based care for sure is going to be based upon the age of the individual and their experience with technology.

Two other interviewees, who were primary care providers, said reading literacy was a barrier, as most programs require participants to read and understand written language on screens. However, as a counterfactual, one mental health provider noted that oversimplifying ISP content might alienate literate individuals:

Any time you are trying to get accessible language that most people understand, you risk inadvertently alienating folks who feel they are being talked down to.

#### Access to the Internet

In 6 interviews (30%, 6/20), patients’ lack of reliable home or community Wi-Fi or hard-wired internet connections were discussed. Where such connections were available, internet speeds may be too slow to support ISP use. Lack of access also included limited bandwidth on cellular phone connections. Interviewees explained that reduced access was influenced by age, personal finance, and locations where cable, cellular, or public networks were not available. One primary care mental health provider said:

For certain members of the older population, lack of consistent, decent access to the internet [is a problem]. I’ve got some guys who are homeless.

#### Internet-Based Self-Care Program Compatibility With Patient Experience

A total of 5 interviewees (25%, 5/20) identified compatibility between patients’ experience of their mental health condition and ISP program content as a determinant of ISP use. Many ISPs deliver versions of manualized therapies, utilizing structured and sequenced formats to teach content and skills for common issues. However, ISPs may not be able to address unique patient experiences. Consequently, some patients may find that ISPs do not align with their specific mental health complaints. A primary care mental health provider shared the following issue:

Sometimes [the patient’s reaction is] like, “Hey, what I’m experiencing doesn’t really fit to this framework.”...I think that sometimes we risk alienating people who feel like we are not appealing to people at their level.

#### Patient Expectations

A total of 4 interviewees (20%, 4/20) discussed unrealistic expectations among patients about the time and effort required for ISP engagement, the application of content to their lives, and symptom improvement. One provider with administrator responsibilities said:

The expectation when you’re on line or using your phone is that things happen quickly. Learning how to relax still takes time. Actually being able to practice and incorporate the techniques still takes time.

### Perceived Determinants Associated With Providers

#### Familiarity With Internet-Based Self-Care Programs

A total of 18 interviewees (90%, 18/20) identified providers’ familiarity with ISPs as a factor influencing use in clinical practice. Familiarity with ISPs ranges from awareness of available programs to regular and judicious use by providers of effective programs for the right patients. Moreover, 10 interviewees stated that they or other providers had limited awareness of any ISPs. One provider with VHA-wide administrative responsibilities stated:

I don’t even think that local staff know what internet-based self-help options out there are.

In addition, 3 interviewees stated that they were aware of programs but were not familiar with their content or had not tried them. One administrator believed that providers must know about all aspects of ISPs to refer patients to them:

As we move forward, the skill of front-line clinical providers is going to be just as much to know the potential benefits, risks, side-affects, value, [and] impact of digital health tools, as they currently know about pharmaceuticals.

For another primary care provider, improving the “findability” of ISPs was key, described as the ability to access the right ISP at the right time for a specific patient.

#### Changes to the Traditional Delivery of Care

A total of 11 interviewees (55%, 11/20) discussed how ISPs could improve current models of care delivery by increasing self-care, providing more patient-generated data, and affording different modes of communication. Interviewees indicated that some providers are reluctant for patients to take part in self-care or treatment outside the traditional provider-patient dyad. One provider who was also an ISP researcher stated:

Some clinicians are reluctant to allow things to happen inside the black box…if the clinician isn’t entirely comfortable with what the rules are, what’s happening inside the app…they’re not going to use it.

Providers were also concerned about how a patient’s clinical status would be monitored and how workload credit for a provider’s time to support ISP use would be tracked. Other interviewees discussed the shift to using patient-generated health data in encounters:

There is a culture shift that needs to happen with providers to help them recognize that patient generated health data has equal value to healthcare system-generated data.

Others focused on possible changes to provider-patient communication, using secure messaging as an example:

Secure messaging impacted their practice. The fear from the provider side was now the Veterans could have an unfiltered access to me and I’m going to spend all day filtering through these e-mails.

#### Competing Demands and Lack of Time

A total of 8 interviewees (40%, 8/20), chiefly primary care and primary care mental health providers, said that competing demands and a lack of time during patient appointments were barriers to ISP use. One said:

It’s just one of many, many things you’re trying to do in the clinic.

Another one said:

I think there would be concern about extra time, like one more thing. More and more keeps getting added on.

Still another provider stated:

Clinicians are not necessarily going to have time to sit there with the patient and show ’em how the thing works, and kinda guide them through it.

### Perceived Determinants Associated With Patients and Providers

#### Human Contact

A total of 10 interviewees (50%, 10/20) discussed human contact as critical for ISP use in primary care settings. One provider who was also an ISP researcher said:

My overall impression is that these are materials that cannot be used without some sort of provider support…I don’t think that sending people to the course or having people download the app is sufficient for those to have any kind of clinical utility.

Interviewees made several points regarding the need to introduce ISPs to patients, elicit interest, assess willingness to engage, and match patients to specific programs. Interviewees also highlighted human contact to support patient progress. One mental health provider said:

I routinely ask, when they come in, “Hey, were you able to use that? What was your experience?” If they didn’t, I try to find out why.

Four interviewees with a range of duties made suggestions about individuals, other than providers, who could support ISP use as a clinical intermediary, such as peer specialists, nurses, care managers, and masters-level clinicians.

#### Age

Age was discussed as a moderator of ISP use for patients in 6 interviews (30%, 6/20) and providers in 3 interviews (15%, 3/20). For patients, interviewees stated that age influences access to the internet, technology literacy, and preference for face-to-face care. One clinic administrator referenced the relatively low use of VHA’s MyHealtheVet website by older Veterans [28]:

I welcome those programs [but] I think it’s challenging. A lot of our Veterans, it’s such an older population, I don’t see a majority of them turning to the internet. I know some will. For example, MyHealtheVet, we thought we’d get a higher population of enrollment for that.

Regarding provider age, interviewees said that the use of technology other than phones for communicating with patients could be a barrier for older providers.

## Discussion

### Study Objective

The aim of this study was to explore primary care provider and administrator perspectives on ISP implementation to identify important perceived determinants of practice for ISP use. We identified 10 themes for determinants of practice at both patient and provider levels. An understanding of these determinants is vital to the development of strategies for ISP implementation in primary care. Comprehensive implementation strategies for ISPs have yet to be clearly defined or rigorously tested. Our investigation of determinants suggests functions for such an ISP implementation strategy, which we feel can be performed by a clinical intermediary (Table 2).

### Internet-Based Self-Care Program Use in Practice

There is little information on rates of ISP use among US providers or within US health care institutions, and we presume this use to be low. Thus, the relatively infrequent use of ISPs reported by this group of interviewees is not too surprising. Nonetheless, interviewees were generally supportive of ISP use, in spite of identifying a number of determinants of practice that can be considered barriers to ISP use. The support for ISPs identified in this study is consistent with the general acceptance of and support for health information technology among providers and patients [13,29]. Support for ISPs relative to actual use may suggest an emerging tipping point for the increased use of ISPs in the United States, as has occurred in other countries. For example, a 2008 survey of mental health providers in the United Kingdom revealed that 99% used ISPs in care and 39% had been trained to use them [30]. At a minimum, our findings indicate that provider and patient attitudes toward ISPs are not strong barriers to implementation.

### Patient-Level Determinants

Perceived patient-level determinants of ISP use including literacy, internet access, and age are well documented, as in a recent analysis of interviews with VHA service users who stopped using an ISP for insomnia before program completion [31-34]. However, conflicting evidence suggests that internet use and the acceptability of ISPs among VHA service users are on par with those of the general population [35,36]. Given these differences and the relative difficulty of modifying the above determinants, compared with other patient-level determinants identified here and in similar studies (ie, interest/motivation, patient expectations, and ISP compatibility with patients), other determinants may be more amenable to mitigation and suitable for incorporation into strategies used to implement ISPs (see discussion below and Table 2) [37].

**Table 2:** Description of how a clinical intermediary can function in an internet-based self-care program implementation strategy to address key determinants of practice.

Determinant of practice		Action of clinical intermediary
**Determinants associated with providers**		
	Provider familiarity	Provide recurring education to providers on ISP^a^ availability and content
	Changes to the traditional delivery of care	Provide supported self-care by mediating communication between the patient and provider regarding clinical questions about the program and progress in the program
	Time/competing demands	Relieve provider workload by sharing responsibility with the provider for case identification, patient education, and program support
**Determinants associated with patients**		
	Patient technology literacy	Perform basic education on program access and navigation such as website location and login. Provide human contact for technical assistance
	Internet access	Identify community internet access points. Develop and maintain clinical internet access points within the health care facility
	Interest/motivation	Supplement the provider in assessing interest in and motivation to engage in behavioral treatment through ISPs using motivational interviewing
	Patient expectations	Supplement provider education about ISPs to ensure realistic expectations on the effort required and time course of symptom improvement
	Patient experience of ISP fit	Provide insight on how program content may fit with an individual’s experience and assist providers in identifying individuals who are failing ISPs or require a higher level of care
**Determinants associated with both patients and providers**		
	Human contact	Meet patients individually or in groups to provide face-to-face support

^a^ISP: internet-based self-care program.

### Provider-Level Determinants

Two themes at the provider level—familiarity with available programs and change to the traditional delivery of care—stand out as key in this study and the few others that have evaluated provider perspectives on ISP use [38,39]. ISP familiarity as a determinant of practice suggests that educating providers about available ISPs and having them experience the platform should be a component of a comprehensive ISP implementation approach. Educational strategies to implement ISPs in health care settings must be flexible to account for varying levels of baseline ISP familiarity among providers. The likely downstream effects of increased provider familiarity with ISPs are the changes more ISP use could bring to the traditional delivery of care [40]. Providers must become more comfortable recommending not only technology use but also self-care to patients. Similarly, a system of stepped care must be in place so that patients who are not appropriate for or fail initial attempts at ISP treatment are identified and referred to other forms of care (eg, more provider interactions) [16].

### A Strategy for Internet-Based Self-Care Program Implementation

The determinants of practice identified here and those from other studies of ISP use outside of primary care, mental health, and VHA settings suggest the need for comprehensive strategies for ISP implementation [31,38,39]. Given the current level of support for ISPs identified among providers in this study, the function of such a strategy should not rely solely on educating providers about ISPs to gain support. Rather, key strategy functions should be more nuanced and include increasing provider familiarity with specific programs, addressing competing demands, providing internet access, and modulating patient expectations, among others (Table 2). Also identified among these determinants is the need for human contact during ISP use. Such contact has already shown evidence for improving adoption and outcomes of ISPs. Support from a human may help address many of the other ISP implementation strategy functions identified here [41,42]. We propose that a clinical intermediary, an individual different from the primary provider, can deliver this human contact. As suggested by interviewees in this study, such a clinical role could be performed by peer specialists, nurses, care managers, and other clinicians trained to facilitate ISP use by engaging in the important implementation strategy functions described in Table 2. Future research should test the use of a clinical intermediary and identify the content, timing, and other specifics of these strategy functions as well as the training needed for this role. In addition, the materials needed to train a clinical intermediary could be spread health system-wide as part of future ISP dissemination efforts.

### Study Limitations

This study has several limitations. Interviews involved a relatively small group of individuals, and the frequency of ISP use within this group was low despite our objective to recruit ISP users. As such, these findings may represent only an initial indication of the important perceived determinants of practice for ISP use, and may be strengthened by a larger sample size or the inclusion of more VHA providers who regularly use ISP in care if they exist. In addition, the purposeful inclusion of individuals who have chosen not to use ISPs in care may reveal yet more critical determinants of practice. Moreover, it must also be noted that determinants were developed from the *perspectives* of VHA providers and administrators. We did not verify these determinants using quantifiable means. However, interviewees had a breadth of relevant backgrounds and adequately represented ISP stakeholders within VHA. They, in turn, discussed a range of determinants of practice that influence ISP use. In addition, interviews with health care consumers may reveal yet more determinants. Organizational-level determinants of practice that might suggest enterprise-wide dissemination and implementation strategies or policy changes were not discussed but will be examined in future work. Finally, the VHA is a unique, integrated, and nationwide health care system that provides care for US Veterans. Thus, our results may not be representative of providers or patients in other health care systems.

### Conclusions

Developing and testing implementation strategies to facilitate the use of ISPs in primary care is a critical area in need of investigation. As an initial step, this study identified patient- and provider-level determinants of practice for the use of ISPs in the context of outpatient primary care settings in the VHA. An ISP implementation strategy that addresses the determinants identified here, including the use of a clinical intermediary to support both patients and providers, may be an effective way forward. These findings, as well as those from similar studies, can be used to inform the development of ISP implementation strategies, which can be further described and tested in future work.

## Abbreviations

ISP: internet-based self-care program
VHA: Veterans Health Administration
